# Supplementation of folic acid in pregnancy and the risk of preeclampsia and gestational hypertension: a meta-analysis

**DOI:** 10.1007/s00404-018-4823-4

**Published:** 2018-07-05

**Authors:** Cheng Liu, Chongdong Liu, Qiushi Wang, Zhenyu Zhang

**Affiliations:** Department of Obstetrics and Gynecology, Beijing Chao-Yang Hospital, Capital Medical University, No. 8 North Road of Workers Stadium, Chaoyang District, Beijing, 100020 China

**Keywords:** Folic acid, Risk of preeclampsia, Risk of gestational hypertension, Meta-analysis

## Abstract

**Objectives:**

We aimed to systematically assess the relationship between folic acid supplementation in pregnancy and risk of preeclampsia and gestational hypertension.

**Methods:**

The relevant studies were included by retrieving the Embase, PubMed and Cochrane library databases. Data extraction was conducted by two investigators independently. The risk ratio (RR) and 95% confidence interval (CI) were used as effect indexes to evaluate the relationship between folic acid supplementation and risk of gestational hypertension or preeclampsia. A subgroup analysis was performed according to the supplementation patterns of folic acid. The homogeneity of the effect size was tested across the studies, and publication biases were examined.

**Results:**

In total, 13 cohort studies and 1 randomized controlled trial study was included, containing 160,562 and 149,320 women with and without folic acid supplementation during pregnancy. Pooled results showed that risk of gestational hypertension was not associated with the supplementation of folic acid. However, folic acid supplementation during pregnancy could significantly reduce the risk of preeclampsia. Moreover, the results of subgroup analysis showed that the decreased preeclampsia risk was associated with supplementation of multivitamins containing folic acid rather than folic acid alone.

**Conclusions:**

Our findings indicate that the supplementation of multivitamins containing folic acid during pregnancy could significantly lower preeclampsia risk.

**Electronic supplementary material:**

The online version of this article (10.1007/s00404-018-4823-4) contains supplementary material, which is available to authorized users.

## Introduction

Gestational hypertension and preeclampsia are common hypertensive disorders during pregnancy [1, 2]. Preeclampsia is a devastating complication of pregnancy responsible for maternal mortality and morbidity [3, 4]. Mothers with preeclampsia during pregnancy may result in neurocognitive dysregulation and suboptimal infant development in offspring [5]. During pregnancy, hypertension is a risk factor for diabetes and cardiovascular disease in later life [6, 7]. Preeclampsia is always caused by impaired placental perfusion, nevertheless, but other risk factors for preeclampsia remain unclear [8].

Accumulating evidences have suggested that elevated levels of blood homocysteine are a cause of gestational hypertension and preeclampsia [9, 10]. In addition, more and more studies confirm that folic acid supplementation can reduce blood homocysteine levels [11, 12]. Furthermore, the relationship between folic acid supplementation and preeclampsia risk has been investigated by several epidemiological studies, but their results are inconsistent. Some studies showed that the supplementation of folic acid is beneficial in decreasing the incidence of preeclampsia and gestational hypertension during pregnancy [13–15]. However, Li et al. demonstrated that there was no association between the occurrence of preeclampsia or gestational hypertension and the consumption of folic acid alone during early pregnancy [16].

In light of these inconsistent results, we conducted this meta-analysis to systematically analyze exploration of the relationship between folic acid supplementation in pregnancy and the risk of preeclampsia or gestational hypertension. This meta-analysis will provide significant clues for further clinical studies.

## Materials and methods

### Search strategy

All relevant published studies were retrieved by searching the databases of Embase (http://www.embase.com), PubMed (http://www.ncbi.nlm.nih.gov/pubmed), and Cochrane library (http://www.thecochranelibrary.com/view/0/index.html) updated to 21 Aug 2017 with the following keywords: pregnancy, folic acid, preeclampsia, and gestational hypertension. The search terms were: (folic acid) AND pregnancy AND {[hypertension OR (gestational hypertension)] OR [pre-eclampsia OR preeclampsia]}. Moreover, manual retrieval for hard copy literature review was also performed in this study for including more studies.

### Inclusion and exclusion criteria

The inclusion criteria were: (1) the study population was pregnant women; (2) comparison group was folic acid supplementation versus folic acid absence; (3) outcome indicators included the risk of gestational hypertension or preeclampsia.

The exclusion criteria were: (1) review literature, conference abstracts, and comments; (2) the studies about the association between the levels of folic acid in serum and gestational hypertension or preeclampsia; (3) animal experiments; (4) gene polymorphism analysis; (5) pregnant women with other specific diseases.

### Data extraction and quality assessment

Data extraction was conducted in all eligible studies by two investigators independently using a standardized protocol. The extracted characteristics included the name of the first author, study design, study period, publication year, demographic characteristics (such as gender and age) of study population, sample size, the supplementation patterns of folic acid, the period of folic acid use, and the incidence of preeclampsia and gestational hypertension After finishing the data extraction, the extraction table was exchanged for check, and the disagreements were settled by discussing. The quality assessments for randomized controlled trials (RCTs) and cohort study were then performed based on the Cochrane risk of bias tool [17] and Newcastle–Ottawa Scale (NOS) [18], respectively. NOS contained 9 points: ≥ 7, 5–6, and < 5 were considered methodologically to be excellent, fair, and poor.

### Statistical analysis

The risk ratio (RR) and 95% confidence interval (CI) were applied as effect indexes to analyze the relationship between the supplementation of folic acid and risk of preeclampsia or gestational hypertension. The homogeneity of effect size across the studies was tested with Cochran* Q* statistics and the *I*^2^ statistics [19]. If *P* < 0.05 and *I*^2^ > 50%, the homogeneity was significant and the random effects model was then applied to pool results, otherwise, the fixed effects model was used [20]. Based on the supplementation patterns of folic acid (folic acid alone versus multivitamins containing folic acid), the subgroup and sensitivity analyses were carried out with Stata 13.0 (StataCorp, College Station, TX, USA). Publication biases were assessed by Egger’s regression test.

## Results

### Description of eligible studies

**Fig. 1:**
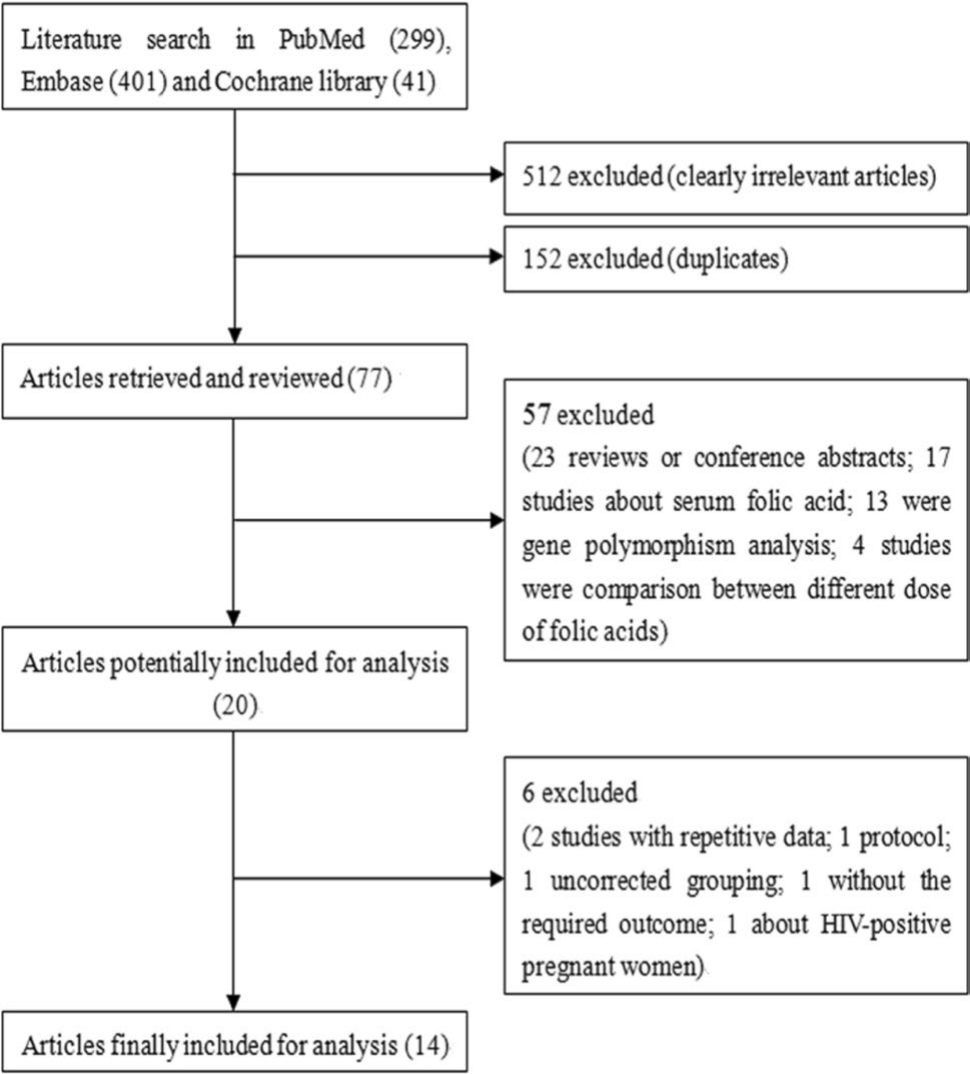
Flowchart of the literature search and study selection

The flow chart for selection of eligible studies is shown in Fig. 1. In total, 741 articles, including 299 in PubMed, 401 in Embase and 41 in Cochrane library, were retrieved. After reviewing the title, 152 duplicates and 512 clearly irrelevant articles were excluded. The remaining 77 articles were then selected by reviewing the abstract, and 57 studies were excluded according to our exclusion criteria, including 23 reviews or conference abstracts, 17 about serum folic acid, 13 gene polymorphism analysis, and 4 about comparison between different doses of folic acids. After further reviewing of the full text, six studies were excluded, including two with repetitive data, one protocol, one uncorrected grouping, one without the required outcome, and one about HIV-positive pregnant women. At last, 14 eligible studies [13–16, 21–30] were included.

The characteristics of included studies are shown in Table 1. There were 13 cohort studies (12–14, 20–22, 24–30) and 1 RCT [24], containing 160,562 and 149,320 women with or without folic acid supplementation during pregnancy. The period in which the women received folic acid was presented: some was in preconception, some were in early second trimester, and some were throughout pregnancy. The included studies were conducted in China, United States, UK, Australia, Denmark, Canada, and South Korea. The NOS assessment showed that the quality of the included 13 cohort studies was excellent (6–9 points). Moreover, RCT conducted by Charles et al. [24] showed that blinding of participants and personnel, allocation concealment, blinding of outcome assessment, and incomplete outcome data were all at the low risk (supplementary Fig. 1).

**Table 1:** The characteristics of included studies

Study	Study type	Country	Study period	The period of folic acid use	Folic acid supplement	No.		Quality assessment
						Suppl.	No suppl.	
Bodnar et al. [21]	PCS	USA	1997–2001	In the past 6 months, daily use	Periconceptional multivitamin use	860	975	7
Bukowski et al. [22]	PCS	USA	1999–2002	In the past 1 year, daily use	Preconceptional folate supplementation	12,444	15,259	7
Catov et al. [23]	PCS	Denmark	1997–2003	4 weeks before the LMP through 8 weeks after the LMP	Periconceptional multivitamin use	21,785	11,503	6
					Folate-only users	2609	11,503	
Charles et al. [24]	RCT	UK	1966–1967	< 37 weeks gestation	Periconceptional folic acid use	1902	917	
Hernandez-Diaz et al. [25]	RCS	USA, Canada	1993–2000	From 2 months before conception through the entire pregnancy	Periconceptional folic acid use	1953	147	7
Kim et al. [26]	RCS	South Korea	2009–2010	NR	Prenatal intake of folic acid	134	81	8
Li et al. [16, 31]	RCS	China	1993–1996	During early pregnancy	Folic acid supplementation	92,731	100,823	9
Liu et al. [27]	PCS	China	2010–2012	Pre- and post-conception	Folic acid intake	7864	2315	7
Martinussen et al. [28]	PCS	USA	1996–2000	First trimester overall	Folic acid supplementation in early pregnancy	3301	346	8
Timmermans et al. [29]	PCS	Netherlands	2002–2006	Before 8 weeks	Preconceptional folate supplementation	2362	1770	9
Vanderlelie et al. [13]	PCS	Australia	2006–2011	The first trimester of pregnancy	First trimester multivitamin/mineral use	719	1066	7
					Folate-only users	476	1066	
Wang et al. [15]	PCS	China	2010–2012	Before conception and/or during pregnancy	Dietary folate intake before conception and during pregnancy	794	265	7
Wen et al. [30]	PCS	Canada	2002–2005	In early second trimester	Prenatal multivitamin use	2317	238	6
					Folate-only users	421	238	
Wen et al. [14]	PCS	Canada	2002–2008	In early second trimester	Prenatal multivitamin use	7265	404	6
					Folate-only users	625	404	

*PCS* prospective cohort study; *RCS* retrospective cohort study; *RCT* randomized controlled trail; *Suppl* supplementation; *LMP* last menstrual period

### Relationship between supplementation of folic acid and risk of gestational hypertension

**Fig. 2:**
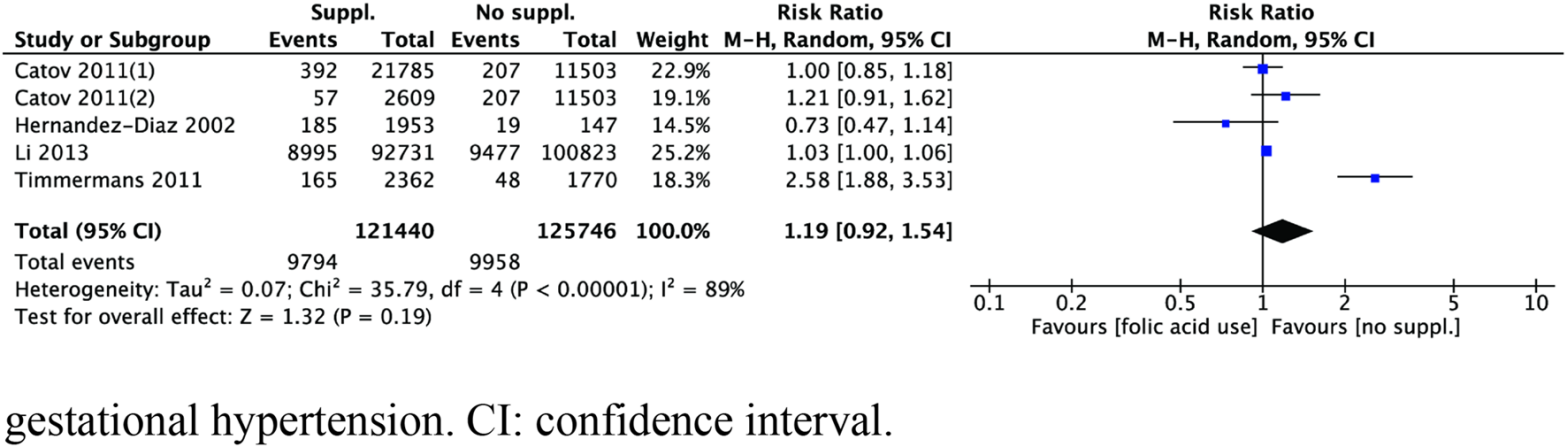
Forest plot of the association between folic acid supplementation and risk of gestational hypertension. *CI* confidence interval

Four studies [23, 25, 29, 31] about the relationship between folic acid supplementation and gestational hypertension risk were included in this meta-analysis. The heterogeneity was high across the above four studies (*I*^2^ = 89%, *P* < 0.01). As shown in Fig. 2, there was no relationship between the supplementation of folic acid and gestational hypertension risk (RR = 1.19, 95% CI 0.92–1.54, *P* = 0.19).

### Relationship between supplementation of folic acid and risk of preeclampsia

**Fig. 3:**
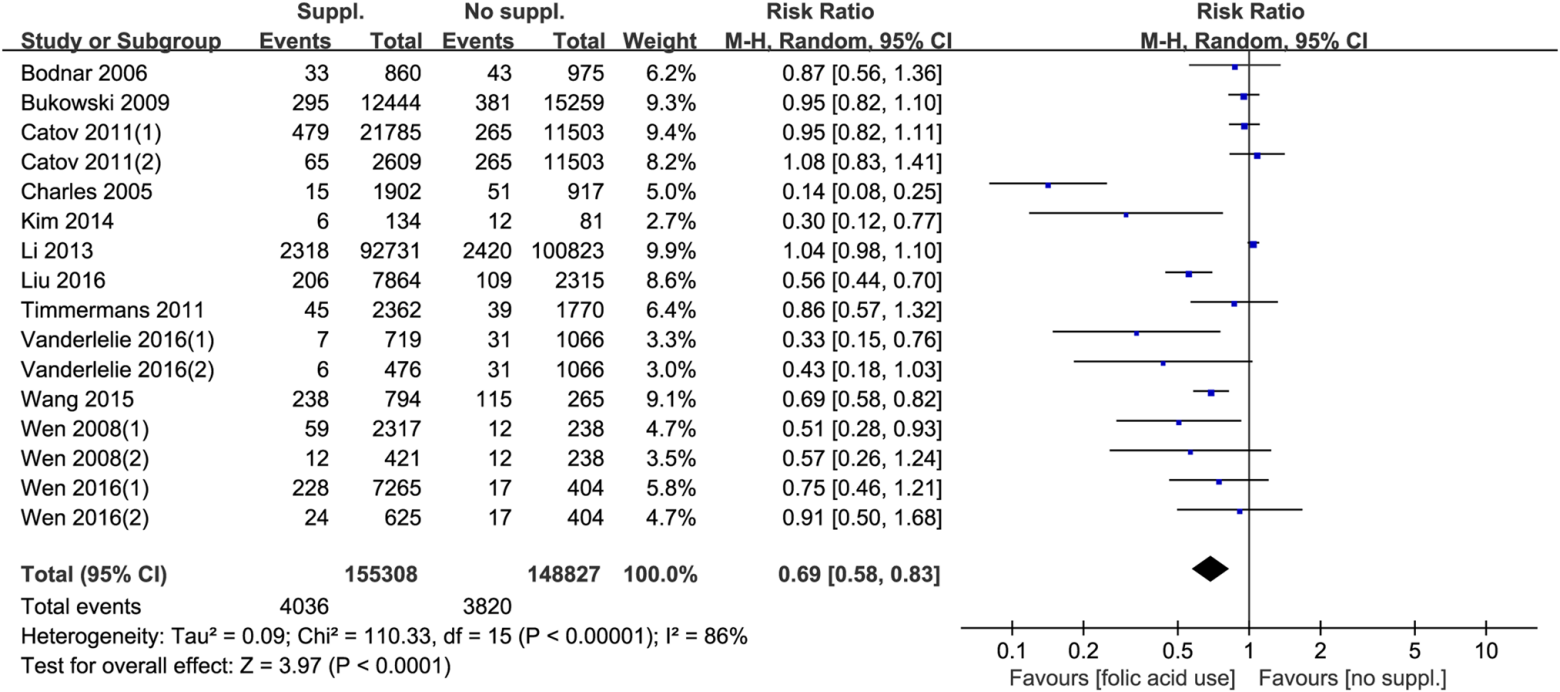
Forest plot of the association between folic acid supplementation and risk of preeclampsia. *CI* confidence interval

Twelve studies [13–15, 21–24, 26, 27, 29–31] were included to explore the relationship between folic acid supplementation and preeclampsia risk. The heterogeneity was also high across these studies (*I*^2^ = 86%, *P* < 0.01), and the pool results showed that the decreased risk of preeclampsia is related to folic acid supplementation during pregnancy (RR = 0.69, 95% CI 0.58–0.83, *P* < 0.01, Fig. 3).

**Fig. 4:**
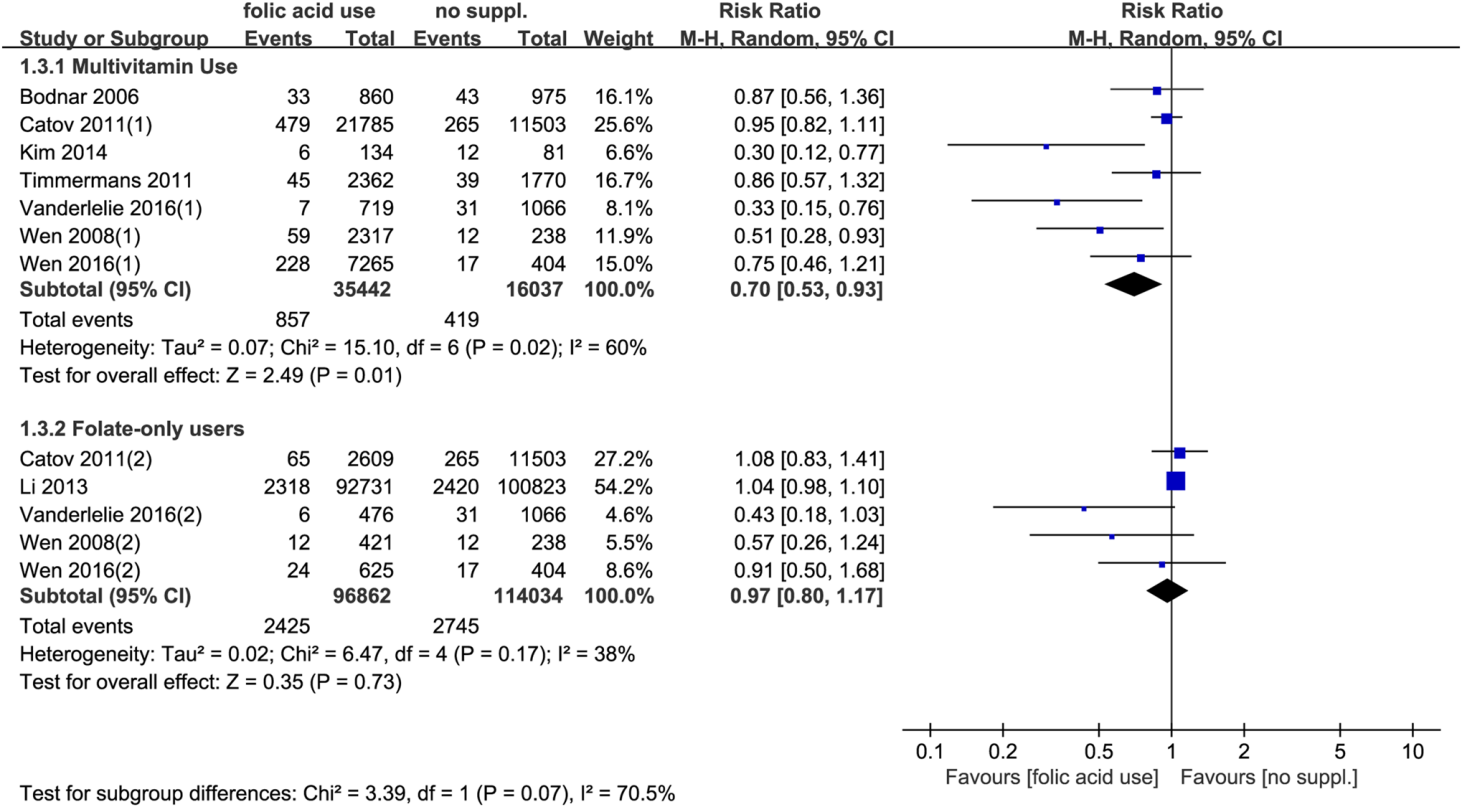
Forest plot of the association between different patterns of folic acid supplementation and risk of preeclampsia. *CI* confidence interval

Furthermore, according to the patterns of supplementation of folic acid, a subgroup analysis was performed. The results showed that the supplementation of multivitamins containing folic acid significantly decreased the risk of preeclampsia (RR = 0.70, 95% CI 0.53–0.93, *P* = 0.01), while the supplementation of folic acid alone had no significant effects on preeclampsia risk (RR = 0.97, 95% CI 0.80–1.17, *P* = 0.73) (Fig. 4).

### Publication bias

**Fig. 5:**
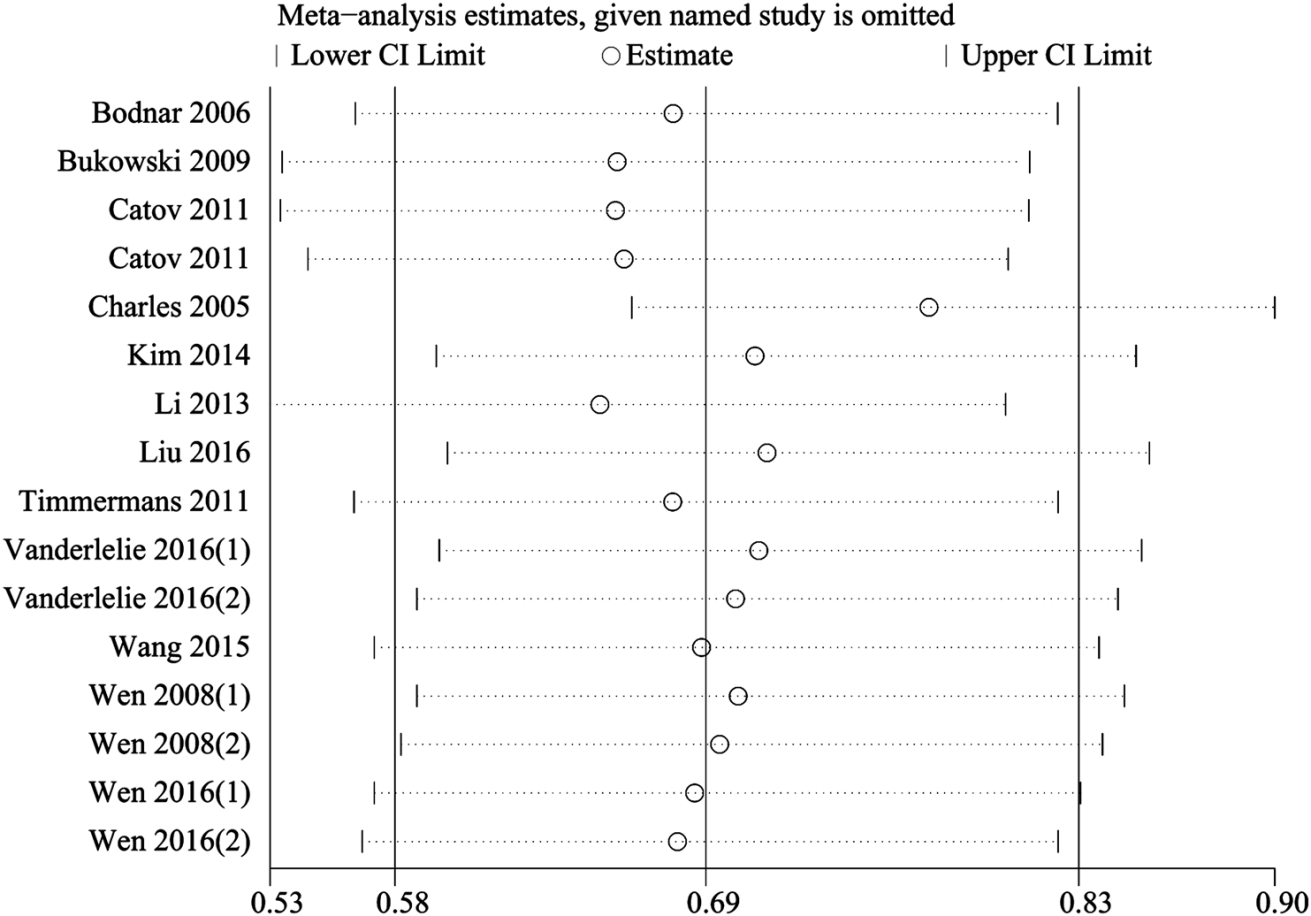
A sensitivity analysis for publication bias of the included studies. *CI* confidence interval

The results of Egger’s test showed that a significant publication bias existed among our included studies (*P* = 0.002). The publication bias was then verified using trim and fill method [32], and the obtained results were not changed. Moreover, a sensitivity analysis was performed by eliminating the included studies one by one, and eventually, the pooled results were also not significantly changed (Fig. 5).

## Discussion

In this meta-analysis, 14 studies were included and pooled results showed that the supplementation of folic acid during pregnancy significantly reduced preeclampsia risk, but had no effects on gestational hypertension risk. Moreover, the decreased preeclampsia risk was associated with supplementation of multivitamins containing folic acid rather than folic acid alone. These results merit further discussion.

Supplementation of folic acid has been found to decrease preeclampsia risk. The possibility is that folic acid can affect the levels of hyperhomocysteinemia, which is suggested to damage the vascular endothelium of the developing placenta [33]. Moreover, a folate deficiency may induce the apoptosis of human cytotrophoblast cells, thus possibly affecting trophoblast invasion and placental development [34, 35]. Therefore, the supplementation of folic acid may improve placental implantation and subsequently affect the incidence of hypertensive pregnancy disorders. In this study, our results are consistent with the findings of a previous study reported by Catov et al. [36] that a decreased preeclampsia risk is related to multivitamin use, but there is no association between the risk of preeclampsia and folic acid supplement alone. Given the biologic rationale of folic acid in reducing the risk of developing preeclampsia, we speculate that folic acid may play a more important role in preeclampsia than other vitamins because the biologic mechanisms of other vitamins in reducing the risk of developing preeclampsia have not been fully explored. Moreover, previous RCTs have found that supplementation with vitamins C and E (without folic acid) during pregnancy has no protective effect on preeclampsia [37, 38]. Lv et al. also demonstrated that the risk for lower clinical pregnancy rate was not significantly correlated with deficient serum vitamin D level in infertile woman undergoing in vitro fertilization [39]. In addition to several studies that have confirmed a relationship between decreased preeclampsia risk and the supplementation of multivitamins containing folic acid [13, 14], we speculate that other vitamins may also play some roles in the prevention of preeclampsia, and the key roles of folic acid in preventing the risk of preeclampsia may be enhanced by other nutrients present in multivitamins. However, the ingredients of multivitamins in different included studies are largely unknown, hindering us to further explore the key other vitamins in preventing the risk of preeclampsia. More studies are still needed to confirm our observation.

Furthermore, strengths and limitations should be considered. Strengths of our study were that a total of 14 articles containing a larger number of patients were included in this study. The larger sample size increased the reliability of our results. In addition, this meta-analysis was conducted on a basis of good quality studies. Although publication bias existed among our included studies, the results of trim and fill method and a sensitivity analysis also confirmed that the pool results had no significant changes. Despite these, our results should be considered in light of limitations. First, a high heterogeneity existed in this study. One possibility for this was that the sample size differed among different studies, which may result in inconsistent 95% CI. Second, among all included studies, 13 were cohort studies and one was a RCT study. Third, we did not collect baseline nutritional data on the cohorts, especially the data on folic acid intake from food. In consideration of the findings of Ray and Mamdani [40] that folic acid from food intake was very low to make any impact on the risk of preeclampsia, we believe that supplementation of multivitamins containing folic acid during pregnancy may be beneficial. Fourth, the dosage of folic acid is largely unknown due to lack of related information in included studies and the period in which the women received folic acid was insistent, which may influence the effects of folic acid on the prevention of preeclampsia. Notably, previous findings showed that there was no significant association between dosage of folic acid supplementation and the strength of its effect [41, 42], but an inverse relationship exists between the duration of folic acid supplementation and the risk of stroke [42], suggesting that the supplementation duration of folic acid is more important than its dosage. A further analysis on exploring key period of folic acid supplementation may have important clinical significance. Considering the different study design among studies, our results are still needed to be further verified by more high-quality clinical studies.

In conclusion, the findings of this meta-analysis indicate that the supplementation of multivitamins containing folic acid during pregnancy may reduce preeclampsia risk. Multivitamin supplementation may be considered as a promising prevention strategy for preeclampsia.

## Electronic supplementary material

Below is the link to the electronic supplementary material.

Supplementary material 1 (DOCX 1918 kb)
