# Supplementary material for: Supplementation of folic acid in pregnancy and the risk of preeclampsia and gestational hypertension: a meta-analysis
Source: Arch Gynecol Obstet. 2018 Jul 5;298(4):697–704. doi: 10.1007/s00404-018-4823-4 (PMC6153594; doi:10.1007/s00404-018-4823-4)
Supplement: Supplementary file 1 — Supplementary material 1 (DOCX 1918 kb) [file 404_2018_4823_MOESM1_ESM.docx]

**Supplementary Figure 1** Risk of bias summary for randomized cont
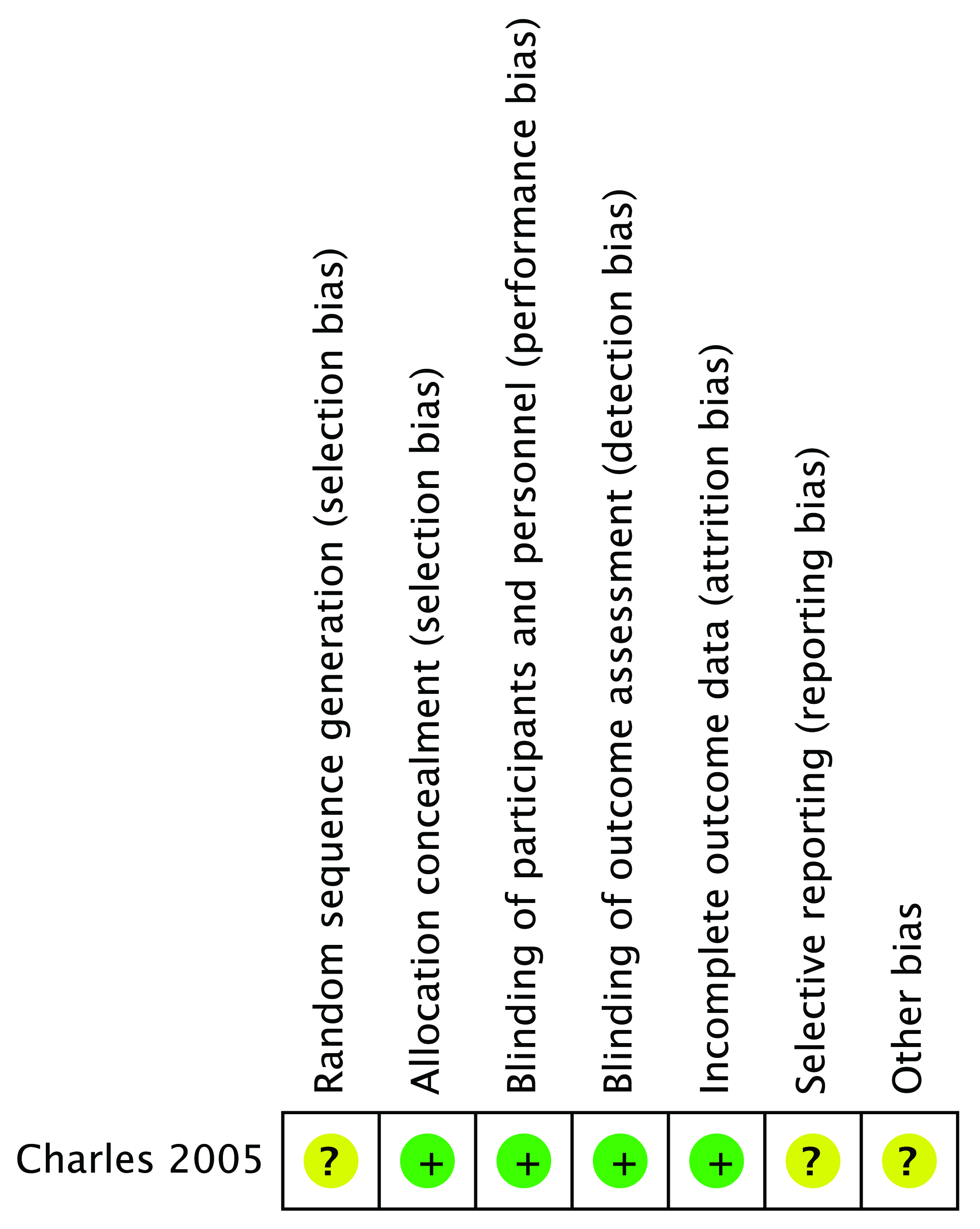
rolled trial conducted by Charles et al.
